# Recovery after Minor Traffic Injuries: A Randomized Controlled Trial

**DOI:** 10.1371/journal.pctr.0020014

**Published:** 2007-03-23

**Authors:** Carin Ottosson, Hans Pettersson, Sven-Erik Johansson, Olof Nyrén, Sari Ponzer

**Affiliations:** 1 Department of Clinical Science and Education, Södersjukhuset, Karolinska Institutet, Stockholm, Sweden; 2 Center for Family Medicine, Karolinska Institutet, Huddinge, Sweden; 3 Department of Medical Epidemiology and Biostatistics, Karolinska Institutet, Stockholm, Sweden

## Abstract

**Objectives:**

To assess the efficacy of an acute multidisciplinary group intervention on self-perceived recovery following minor traffic-related musculoskeletal injuries.

**Design:**

Open, randomized controlled trial.

**Setting:**

A large inner-city hospital.

**Participants:**

127 patients (≥15 y) with traffic-related acute minor musculoskeletal injuries and predicted to be at risk for delayed recovery were randomized into an intervention group (*n* = 65) or a control group (*n* = 62).

**Intervention:**

Four 1½-h sessions in open groups with the aim of providing information about injuries in general, calling attention to the importance of self-care and promoting physical activity. In addition, both groups received standard medical care by regular staff.

**Outcome measures:**

The main outcome measure was self-reported recovery at 12 mo. Secondary outcome measures were ratings of functional health status (SF-36, SMFA), pain and mental distress on visual analog scales, and self-reported duration of sick leave.

**Results:**

At 12 mo, there was a 21.9 percentage point difference: 52.4% of the patients in the intervention group and 30.5% in the control group reported self-perceived recovery (95% confidence interval for the difference 5%–38%; *p* = 0.03). There were no statistically significant differences between the groups regarding the secondary outcome measures.

**Conclusion:**

A simple group intervention may accelerate the self-perceived recovery in selected patients. As we did not find evidence of improvements in the secondary outcome measures, the clinical significance of the treatment benefit remains to be defined.

## Editorial Commentary

**Background:** Worldwide, road traffic accidents contribute substantially to the number of deaths and also to the burden of disability. However, there is a lack of research into road traffic accidents as compared to other causes of ill-health. In particular, minor injuries resulting from traffic accidents are not well-studied even though some people with such injuries might be unwell for a long period of time. Support programs that provide people who have had minor traffic-related injuries with psychological help, physical training, and other types of interventions might help people to recover more quickly. However, there is little evidence that would help to answer this question. In the trial reported here, the researchers aimed to find out whether a support program would increase the chance of recovery in people who had experienced minor traffic-related injuries and who were thought to be less likely to recover. Trial participants were randomized to receive either standard medical care or to receive standard care and also to attend a series of workshops where surgeons, psychiatrists, pain specialists, and other staff gave advice about healing, pain management, exercises, and other aspects of self-care. The primary outcome of the trial was whether participants considered themselves to have recovered, 12 mo after the injury.

**What the trial shows:** 127 patients were recruited into the study, 62 of whom were assigned to receive standard care only and 65 of whom were assigned to also attend the support workshops. Most participants assigned to the support arm did attend all of the workshop sessions. Patients assigned to attend the support workshops were more likely to report that they had recovered as compared to patients receiving standard care only, and this difference seemed quite substantial. However, there were no statistically significant differences between these two groups in the trial's secondary outcome measures. These secondary measures included scores on rating scales which attempt to measure physical and mental distress and coping ability and the amount of sick leave taken.

**Strengths and limitations:** Although the trial was fairly small, it did recruit enough participants to detect an effect in the primary outcome measure, recovery at 12 mo. Additionally, a large proportion of the participants randomized to attend support sessions did actually attend the sessions, and follow-up for the primary outcome measure was virtually complete. Limitations of this study include low follow-up of patients for the secondary outcome measures in the trial. This meant that there was low power to detect clinically relevant changes in these outcome measures. Finally, although many patients were eligible for the trial, only a small proportion could be successfully contacted after being discharged from hospital, and it is possible that those people who did agree to participate were more motivated to recover than patients in general who have these injuries.

**Contribution to the evidence:** Most evidence relating to the benefits of support programs such as these have evaluated their effects in patients who have whiplash injuries rather than general traffic-related injuries. However, the results from this trial are compatible with those of similar trials in patients with whiplash injuries and suggest that support programs such as these may be beneficial in patients at risk of not recovering well from their injuries.

## INTRODUCTION

Minor traffic-related injuries, including whiplash type of neck injuries, are common and may result in unexpectedly protracted disability [1]. While whiplash-associated disorder (WAD) has attracted considerable scientific attention [2–4], other minor traffic-related injuries have been less well studied**.** However, some studies suggest that other minor injuries may have a similar prognosis as WAD [5–7].

Hence, among patients with seemingly trivial injuries, there are individuals with a high risk of substantially delayed recovery. The reasons suggested for a slower recovery, or even for permanent disability, include factors other than purely injury-related ones.

It has also been suggested that psychosocial and other support programs, given in addition to the correct medical and surgical treatment and rehabilitation, might enhance recovery after traffic injuries. This additional support has been provided in several ways and forms but has almost exclusively been offered to WAD patients [8,9]. Provinciali et al. showed that a multidisciplinary program consisting of postural training, manual technique, and psychological support had a significant effect on the time to return-to-work in WAD patients [8]. In a study by Vendrig et al. [9], it was shown that multidisciplinary treatment with physical training, graded physical activity, occupational therapy, and pain behaviour therapy was effective for patients with chronic WAD symptoms. The positive effects of multidisciplinary treatments in WAD patients have also been confirmed in meta-analyses by Verhagen et al. [10] and Sefriadis et al. [11].

We hypothesized that in patients with risk factors for protracted recovery and suffering from minor traffic-related musculoskeletal injuries, a multidisciplinary group intervention with information about the injury, pain management, activity level, and support of healthy instead of illness behaviour during the acute phase of rehabilitation, might shorten the time to recovery [8,11,12].

The objective of this study was to assess the efficacy of such an intervention in patients with acute traffic-related minor musculoskeletal injuries, rated to be at high risk for delayed recovery, based on the scores obtained in a newly developed prediction ruler (prediction of prolonged self-perceived recovery after musculoskeletal injuries, the PPS). The primary endpoint was the patients' self-perceived recovery after 12 mo.

## METHODS

### Participants

Potentially eligible patients had sustained traffic-related minor musculoskeletal injuries (Injury Severity Score <9) [13] less than 24 h before arrival to the emergency department (ED) of a large inner-city hospital in Stockholm, Sweden. Exclusion criteria were a major musculoskeletal injury (Injury Severity Score ≥9) [13], age ≥15 y, inability to read and understand Swedish, or impaired cognitive function as judged by the investigators. Consecutive patients were evaluated, but to be eligible for randomization the patients had to have a high risk of prolonged recovery according to the PPS questionnaire [14]. The PPS is a novel instrument for prediction of non-recovery 4–6 mo after a musculoskeletal injury and consists of four questions to be answered by the patient (working status, educational level, and ratings of injury-related pain and mental distress) and a rough injury classification (neck pain after a whiplash type of injury, contusion, dislocation/distortions, or fracture) provided by the staff. We have shown the PPS predicts subjective non-recovery after 6 mo with greater accuracy than predictions based exclusively on information about the injury. The study was approved by the local Ethics Committee and all patients gave their informed consent before inclusion.

### Intervention

Eligible patients were randomised to an intervention or a control group. Both groups received standard medical treatment according to the routines at the department. The intervention offered was based on principles discussed by Fordyce [15], Linton [16], and Vendrig [9], but was adapted for an acute injury population through several workshops with experts in orthopedic surgery, psychiatry, cognitive psychology, anesthesiology/pain treatment, social medicine, epidemiology, physiotherapy, and nursing. The intervention was designed to be simple and clinically practicable and consisted of four group sessions, each lasting for approximately 1½ h, once weekly, in open groups. The intervention aimed to supply generic information about tissue healing after injuries and about pain management, to call attention to the importance of self-care, and to propose exercises in relaxation and postural control [17]. After a first introductory session (given every week), to which the intervention group was invited within 1 wk after the injury, the subsequent three sessions, held sequentially in 3-wk cycles, were led by the study physiotherapist, the study anaesthesiologist, and the team psychologist, respectively. After having attended the introductory session, new patients entered the cycle with the session that happened to be next. During the sessions, the participants were encouraged to share their experiences with each other. The session leaders acted as group facilitators and also met regularly to coordinate their activities.

### Objectives

This open, randomised controlled clinical trial compared standard treatment with standard treatment supplemented by a multidisciplinary group intervention.

### Baseline Data

Baseline data included information regarding age, sex, injury type (PPS variable), educational level (PPS variable), working status (PPS variable), current diseases, and previous traffic injuries and was collected in connection with the inclusion at the outpatient department before randomisation. Retrospective ratings (i.e., the patients were asked to consider the week before the injury) were carried out to detect signs of anxiety and depression using the Hospital Anxiety and Depression Scale (HAD) [18,19] or signs of posttraumatic stress syndrome (PTSD-10) [20]. We further asked the patients to rate their functional health status (SF-36 [21]) and level of physical functioning using the Short Musculoskeletal Function Assessment (SMFA) [22,23] the week preceding the injury. Finally, the patients were asked also to rate their current physical (“Rate the level of your injury-related physical discomfort/pain”) and mental (“Rate the level of your psychological discomfort/feelings of depression or anxiety”) distress (both PPS variables) as well as their coping [24–26] capability (“How well do you think you can handle your current situation?”) on visual analog scales.

### Outcome Measures

The primary outcome measure was the patients' self-perceived recovery at 12 mo measured by the single question “Do you feel recovered after the injury?” (Yes/no). Secondary outcome measures were the SF-36, the SMFA, the visual analog scales ratings regarding physical and mental distress and coping ability, as well as self-reported duration of sick leave. The patients were followed up by a mailed questionnaire, and if no answer was received the patient was contacted by phone by the study nurse and asked to report whether he/she felt recovered or not.

### Sample Size

The sample size was calculated to detect a difference in the proportions for the primary outcome measure between the control and the intervention groups of 30 percentage points (specifically 30% versus 60%) at the 12-mo follow-up. A total of 49 patients in each group (98 patients) were required to detect this difference with 80% power at 5% significance level, two-tailed. In addition, we anticipated a dropout rate of 25% and the recruitment goal was therefore determined to 140 patients.

### Enrollment, Randomisation, and Blinding

The patients were identified and informed about the study by the study nurse either while still at the ED or via daily ED listings of admitted patients. Patients who had been discharged were contacted by the study nurse by phone, and if no answer was received, an invitation letter was mailed to the patient. For consenting patients, an outpatient appointment was booked at the orthopaedic out-patient department within a week, whereupon each patient with a high risk of prolonged recovery, according to the PPS questionnaire, was allocated to either of the two treatment arms. Randomisation was accomplished through the opening of sequentially numbered, sealed opaque envelopes (160 envelopes). These envelopes contained information about treatment assignment, which was arranged by staff not involved in the study. The practical arrangements for treatment implementation were made immediately following the randomisation. As blinding of the intervention was impossible, the trial was open but the patients were kept blinded to the results of the PPS prediction.

### Statistical Methods

Fisher's exact test was used to compare the intervention and control groups with regard to the proportion of patients reporting self-perceived recovery at 12 mo (i.e., the primary outcome measure). We used the two-sample *t*-test and the chi-square or Fisher's test to compare the groups regarding baseline characteristics and the secondary outcome variables.

The results were considered as significant if *p* was less than 0.05, two-tailed. The *p*-values are presented without any adjustment for multiple comparisons. We present the corresponding 95% confidence intervals (CI) for the differences between the proportions and means with calculations based on the normal approximation. All analyses were performed according to intention-to-treat (ITT) principle. Statistical analysis was performed using the SPSS version 13.0.

## RESULTS

### Participant Flow and Recruitment

**Figure 1:**
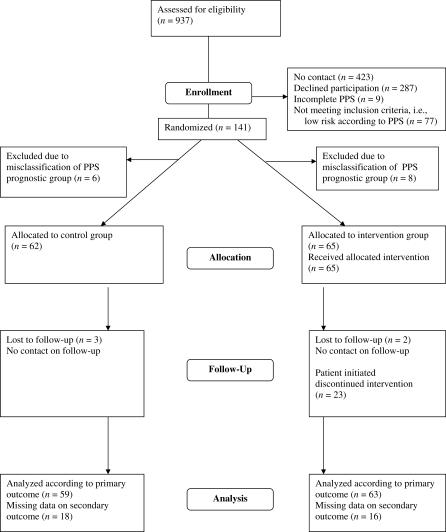
Flowchart

Patients were recruited from September 2002 through January 2004. Of the 937 consecutive patients assessed for eligibility, no contact was established due to no answers to telephone calls or mailed contact letters in 423 (45%) cases; 287 (31%) declined participation; and nine (1%) patients had an incomplete PPS and therefore could not be included. Thus, of the 218 (24%) patients who consented to participate, the PPS classified 77 as having a low risk of non-recovery, and they were therefore excluded in accordance with the study design. Of the remaining 141 patients, 73 were randomised into the intervention group and 68 into the control group. A total of 14 patients were withdrawn since they had been misclassified by the computer program as belonging to the high-risk stratum when, in fact, they belonged to the low-risk group according to the PPS, six belonging to the control group and eight to the intervention group. Therefore, they were not eligible for randomisation. Consequently, a total of 65 patients were allocated to the intervention group and 62 to the control group (Figure 1).

Complete follow-up data assessed from September 2003 through January 2005 were available for 75% (49/65) of the patients in the intervention group and for 71% (44/62) in the control group. With addition of an abbreviated telephone interview regarding the primary outcome measure the corresponding figures were 97% (63/65) and 95% (59/62), respectively.

Of the 65 patients in the intervention group, 65% (42) participated in all four intervention sessions, 12% (8) attended three sessions, and 23% (15) attended two or fewer sessions.

### Baseline Data

**Figure 2:**
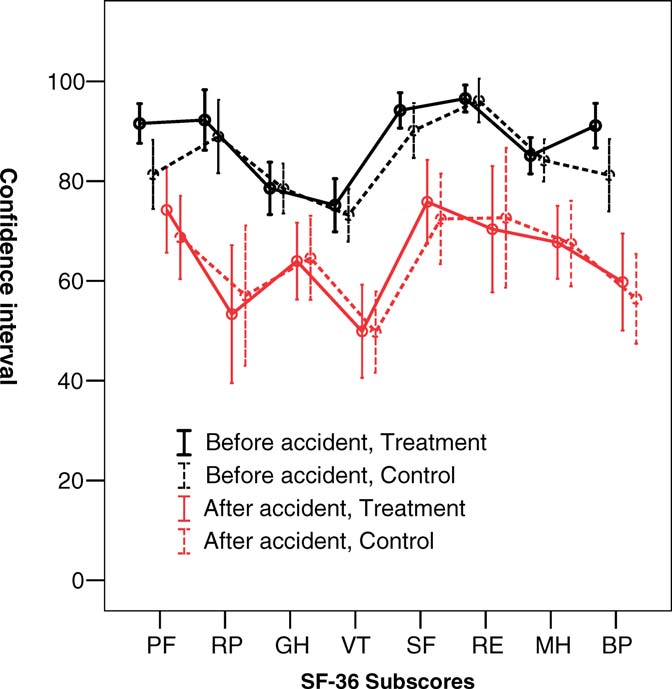
The SF-36 Subscores at Inclusion and at 12-mo Follow-Up The upper black lines represent the 95% CI for the mean for the subscores at inclusion, and the lower red lines represent the subscores at 12-mo follow-up. The solid lines represent the ratings of the intervention group and the dotted lines the ratings of the control group. The x-axis represents the different dimensions of the SF-36: physical functioning (PF), limitations in usual role activities due to physical health problems (RP), bodily pain (BP), general health (GH), vitality (VT), social functioning (SF), limitations in usual role activities due to emotional problems (RE), and mental health (MH).

The intervention and control groups were found to be comparable, regarding background variables, including signs of depression/anxiety (HAD) and PTSD-10 (Table 1). The groups did not differ at baseline regarding visual analog scale ratings of pain or mental distress, but there was a slight difference regarding the SMFA bothersome index between the groups (Table 2). There were no significant differences regarding subscores of the SF-36 except for physical functioning with the intervention group having a slightly better physical functioning score compared to the control group (Figure 2).

**Table 1:**
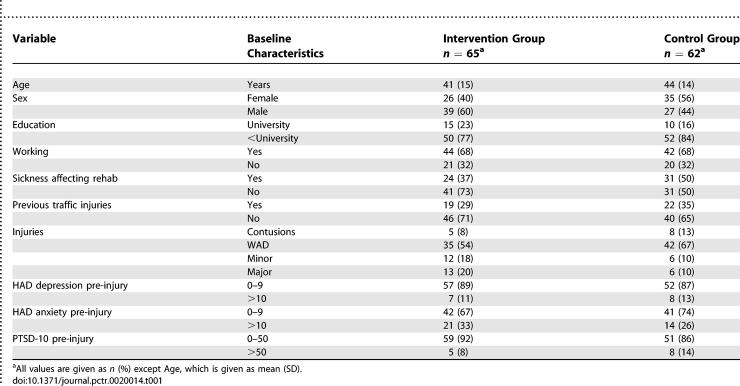
Baseline Characteristics of the Study Population (No Significant Differences between the Intervention and the Control Groups)

**Table 2:**
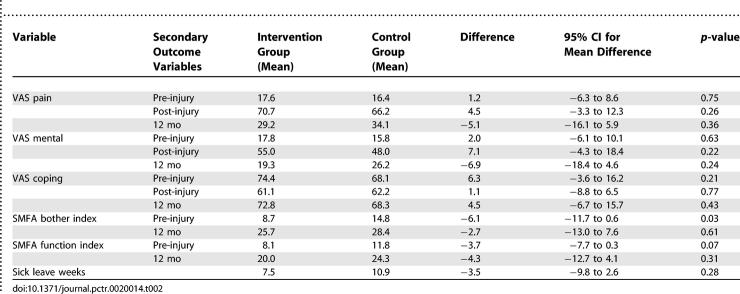
Secondary Outcome Variables at Baseline and at 12 mo for the Intervention and for the Control Groups (No Significant Differences between the Groups)

### Outcomes

At the 12-mo follow-up, 52% (33/63) of patients in the intervention group and 31% (18/59) of the patients in the control group with known outcome reported recovery (difference 0.22, 95% CI; 0.05–0.38, *p* = 0.03). Further analyses of the dropouts did not change the significant findings (Table 3).

**Table 3:**
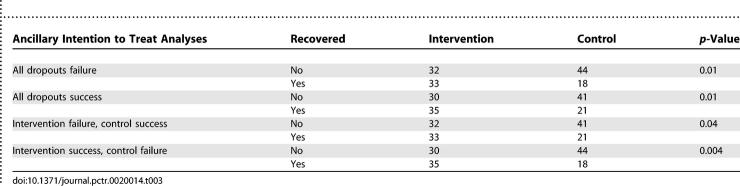
Intention To Treat Analyses

No significant differences were found between the groups regarding ratings of physical or mental distress, coping ability, or the SMFA (Table 2). The SF-36 results (Figure 2) were also comparable between the groups. The mean duration of self-reported sick leave was 7.5 wk (SD = 15.5) in the intervention group and 10.9 wk (SD = 17.5) in the control group (difference; 3.4 wk, 95% CI; −2.9 to 9.8, *p* = 0.18).

### Ancillary Analyses

A total of 54 patients of the 93 available reported an overall frequency of return visits (orthopedics, primary health care, or private practitioner) at 12 mo with no differences between the intervention and control groups. During the follow-up, 24% (12/49) of the patients in the intervention and 14% (6/43) of the patients in the control group reported a new injury or a disease of which eight in the intervention group and four in the control group were considered to affect their rehabilitation from the previous injury (difference 0.11, 95% CI; −0.05 to 0.26, *p* = 0.70).

## DISCUSSION

### Interpretation

This study assesses the efficacy of a multidisciplinary intervention aimed at speeding up recovery in patients with acute minor musculoskeletal injuries predicted to be at high risk of delayed recovery. At the 12-mo follow-up the intervention group reported recovery (the main outcome measure) significantly more often than the control group did. Our results can therefore be regarded as encouraging, but as we were unable to statistically confirm improvements in the secondary outcome measures the clinical significance of the treatment benefit remains to be defined.

A major limitation of this study was that we were able to include only about one fourth of the eligible patients. The reasons for non-participation in the majority, however, were of administrative nature since we could not get in contact with about half of the patients entering the ED. Only one fourth of the non-participants actively declined participation. Even if there is a risk of bias it is reasonable to assume that less than all patients with minor injuries would be interested in any rehabilitation and some might be persons out of reach for any intervention, as suggested by Cutler et al. [27], implying that self-selection may have enriched our study of patients who were more motivated than average. It is, however, reasonable to assume that the same self-selection will also occur if this intervention will be deployed in routine care.

There are some possible explanations for the lack of significant findings regarding the secondary outcome measures. The first and the most obvious is lack of power; the power calculation was based on the primary outcome with a 30% of difference between the groups. A power calculation for clinical important differences for the secondary outcomes, for example, SF-36, would have yielded a bigger sample size. Second, there are differences between people's interpretation of recovery, and even if not measurable in terms of functional outcome or health-related quality of life, the intervention might have changed this interpretation toward more acceptance of residual symptoms, impairments, or disabilities, as suggested by Beaton [28]. Third, about 30% of the patients in the intervention group did not participate in all of the four sessions, which might have diluted the effect of the intervention in terms of functional outcome. Fourth, we were able to get complete follow-up data, i.e., the secondary outcome measures, for only about 70% of all patients. On the other hand, the intervention group had a non-significant tendency toward shorter sick leave duration compared to the control group, implying that this type of intervention in the early stage after an injury might have long-term effects also in terms of working capability.

### Generalizability

One of the strengths of this study was that the intervention program was only offered to patients at increased risk of non-recovery as predicted by the PPS questionnaire [14]. The rationale for this approach was supported by earlier findings indicating that many patients seem to be able to cope with any injury without specific support and that unnecessary treatment might augment their feeling of not being well [29]. We have previously shown that the PPS can predict an unfavorable outcome with a better accuracy than predictions based exclusively on information about the injury. Even if the PPS has only fair sensitivity and specificity it is the first step toward targeting the patients in need of extra support.

Another of the strengths is that the intervention was aimed to be clinically applicable and was therefore short and gave the patients flexibility to choose dates for their participation. Over 75% participated in at least three of the four possible sessions, indicating that this goal was mainly reached. The intervention was based on well-known principles and uncontroversial per se. Besides the content of the sessions, the participation itself probably promoted the patients' physical activity level, and the discussions during the sessions were most likely of importance as a means of applying a certain degree of social pressure, inter-participant support, and encouragement. Another important feature of our intervention program was the possibility for continuous contact with the study personnel, an important factor in any medical treatment.

In conclusion, this study provides some evidence that among selected injured patients with constellations of factors that are indicative of a high risk of a slow return to self-perceived health, a simple group intervention can stimulate recovery. Our intervention, focused on helping the patient appreciate the healthy parts of their injured body and mind and stressing the importance of physical activities already during the early phase of the rehabilitation, should be possible to replicate by others. As our intervention was comparatively simple and moderately demanding, both in terms of health care resources needed and time expenditure on the part of the patients, it tentatively appears to be practicable and economically justifiable in routine care. At least the results are encouraging enough to warrant further studies.

### Overall Evidence

Despite the common occurrence of minor traffic injuries [30], few studies have addressed the effects of auxiliary supportive measures in consecutive patients with a broad spectrum of traffic-related injuries. Most studies of such interventions have concerned specified conditions such as WAD [4]. The only exception, to our knowledge, is a study of debriefing in patients with mixed minor traffic injuries [31]. This study found a worse than average outcome among patients who received the active intervention, compared to those in the control group. Two principal explanations have been proposed for this unexpected result: first, as the treatment was applied to all patients, including those with an already good prognosis, it is conceivable that unnecessary treatment might have augmented a feeling of not being well in a non-negligible proportion. Second, others have pointed out that debriefing was developed for planned stressful events. Therefore, debriefing might not be effective after unexpected accidents.

The only randomised controlled study of multidisciplinary treatment that we could find in the literature was performed by Provincali et al. [8]. They randomly allocated 60 WAD patients to either an experimental multimodal treatment consisting of postural training, manual technique, and psychological support, or to a control treatment with physical agents only, such as electrical and sonic modalities. They found significantly better and more long-lasting improvements among the patients who received the experimental treatment. Although this study, as opposed to ours, was restricted to WAD patients and the control condition involved a specific treatment, the observed effect, albeit surprisingly strong, is consistent with our overall result.

Other studies, however nonrandomised, of multidisciplinary treatment of chronic WAD [32], suggest that such intervention might be beneficial, with significant reductions in pain among actively treated patients. Apart from the possibility that some of the effect in these observational studies could potentially be attributed to confounding, the interventions were generally much more time and resource consuming than that used in our study.

Other studies among patients with WAD have focused on specified supportive measures rather than on multidisciplinary treatment. Ferrari et al. [33] were unable to confirm any benefit from an educational pamphlet on outcome. On the other hand, Brison et al. [34] used an educational video as intervention and found a trend toward less severe WAD symptoms. A systematic review by the Cochrane collaboration concerning treatment of WAD [4] found early mobilization to be effective. Early mobilization was also a part of our intervention. The Cochrane review further concluded that studies of multidisciplinary treatment strategies are warranted.

Future studies should focus on the clinical significance and cost effectiveness of this intervention. In particular, future studies should be dimensioned so that they are able to confirm clinically important effects in our secondary outcome variables, notably duration of sick-listing and quality of life. Careful measurements of costs, from the perspective of the individual, health care, and society would also be a valuable addition. Further development of the clinical prediction rule, the PPS, still admittedly crude, is also warranted. It is conceivable that a better classification of patients in terms of prognosis will improve the selection of suitable cases and thus enhance the efficiency of the intervention. Finally, additional fine-tuning of the contents in the multidisciplinary intervention, with randomised evaluations of the single components, might improve its performance even more.

## SUPPORTING INFORMATION

CONSORT Checklist Click here for additional data file. (50 KB DOC)

Trial Protocol Click here for additional data file. (42 KB DOC)

## Abbreviations

CI: confidence interval
ED: emergency department
HAD: Hospital Anxiety and Depression Scale
PPS: prediction of prolonged self-perceived recovery after musculoskeletal injuries
PTSD-10: post-traumatic stress syndrome
SD: standard deviation
SFMA: Short Musculoskeletal Function Assessment
WAD: whiplash-associated disorder
