# Supplementary material for: Recovery after Minor Traffic Injuries: A Randomized Controlled Trial
Source: PLoS Clin Trials. 2007 Mar 23;2(3):e14. doi: 10.1371/journal.pctr.0020014 (PMC1829405; doi:10.1371/journal.pctr.0020014)
Supplement: Trial Protocol [file pctr.0020014.sd002.doc]

# Psychosocial support and minor traffic injuries – a randomized controlled intervention study

The aims of the study

This randomised controlled study aims to investigate the effect on recovery rate of a multidisciplinary support program for patients injured in traffic and at risk of non-recovery.

Background

It has been estimated that traffic injuries account for about 13% of all injury-related costs and that minor injuries are generally responsible for a large part of the injury-related burden for society. Most traffic victims seen in orthopaedic emergency rooms suffer from minor or moderate injuries, and only a minority of them requires hospitalization. Since the need for qualified orthopaedic treatment is usually of a limited nature, patients with minor injuries have received comparatively little attention both in clinical care and in medical research. Long-term disability after whiplash neck injuries is a well-known phenomenon meanwhile it is less well established that many patients with other traffic accident related minor injuries also have a slow recovery and that long-term adverse psychiatric and social consequences are common.

Our recent study focusing on 1- and 6-month outcomes for traffic accident victims showed that among the 318 patients included, self-perceived recovery was reported by 31% and 56% at 1 and 6 months, respectively. With control for mutual confounding in a multivariate model; injury severity, working status and educational status were associated with 4.5 fold, 3.2 fold and 2.3 fold respective gradients in odds of not feeling recovered at one month. At 6 months only working status (OR=3.2) and educational status (OR=2.3) were associated with the risk of non-recovery. The 6-month prognosis of whiplash neck injuries was similar to that of other injuries. Self-perceived non-recovered patients reported more pain and anxiety than self-perceived recovered patients. Similar results have been found in studies among other trauma victims: patients with a less good outcome more often have a lower educational level and a higher unemployment level with more previous psychosocial or psychiatric problems.

Even if several authors have demonstrated the importance of psychosocial factors in the recovery process after trauma there are only a few randomized studies on psychosocial support. Our group has in a previous randomized study on a mixed orthopaedic injury population shown that an individual psychosocial support program has a positive effect on health-related quality of life (HRQoL). However, an individual program is money and time consuming and is hard to implement in a regular hospital setting. Further, all patients are not in need of this kind of support and therefore the resources should be focused on patients that might benefit from them. Based on our previous (pooled) data we have developed a simple instrument (questionnaire), preliminary called the PPS-Risk (Prediction of Psychosocial risk factors in acute trauma). The PPS Risk seems to identify patients with a less good prognosis regarding recovery after injuries.

There are several treatment options for patients with chronic musculoskeletal disorders. Multidisciplinary intervention including cognitive behavioral therapy, physiotherapy and information in small groups has had good results among patients with long term problems after whiplash injuries. As far as we know there are no prospective studies focusing on early identification (first 2-8 weeks after injury) of injured patients at risk of non-recovery and offering them a multidisciplinary rehabilitation support program. We believe that such an effort is of major importance since it might offer a possibility to prevent long-term suffering for the individuals at risk.

**Material and Methods**

Patients treated in the Accident and Emergency Department at Söder Hospital, Stockholm for minor acute orthopaedic injuries caused by traffic accidents are eligible for this study. Those aged below 15, non-Swedish speaking, resident outside our catchment area and those with severe injuries (Injury Severity Score >9) area are not eligible. All patients receive medical/surgical treatment according to existing hospital routine. The patients are contacted by a research nurse, by telephone and/or post, within one week after the accident irrespective of whether the patient is admitted or discharged.

If the patient agrees (informed consent) to participate in the study, an appointment at the outpatient department is arranged with the study doctor and nurse. At this time the following baseline data is collected:

- Background data (questionnaire): age, gender, socioeconomic data, smoking and alcohol habits, previous / present morbidity
- Injury related data (medical records): diagnosis, injury type, treatment and planned follow-up visits
- Health related quality of life (questionnaires): SF-36 (generic, profile) and SMFA (disease specific).
- Ratings (Visual Analog Scale, VAS): pain, psychological well being, sense of control over the situation
- Psychological evaluation (questionnaires): Posttraumatic stress syndrome (PTSS 10), depression (Hospital Anxiety and Depression scale, HAD), Coping strategy (Sense of Coherence, SOC)

All questionnaires above are validated and have been used in Swedish populations.

All patients fill in the PPS Risk questionnaire and those at risk according to the prediction model are randomized to the intervention or to the control group (sealed envelopes). Patients without risk factors according to PPS Risk form the control group for the prediction.

*Intervention (Multidisciplinary treatment)*

A team consisting of an orthopaedic surgeon (study doctor), an anaesthetist specialized in pain treatment, physiotherapist, study nurse and a psychologist carry out the intervention program consisting of group “lectures” where different topics are highlighted. The aim of this “Traffic injury school” is to, in a systematic and supportive manner; provide an information and discussion forum for topics that are relevant to all study patients regardless of the type of injury. The program consists of 4 short (about 2 hour) sessions once a week during a 4-week period. The first lecture consists of an introduction to the program and a discussion about different coping mechanisms. The following 3 lectures deal with issues related to pain and pain treatment, the importance of physical training, how fractures and soft tissue injuries heal, and the interaction between mental and physical health. All sessions are lead by the study doctor, anaesthetist, physiotherapist or psychologist and the study nurse participates in all sessions. After the introduction session the patients may participate in consecutive sessions or they can choose to come to later sessions as the groups are “open” and the “school” runs on a 3-week rolling schedule. After every session the patients rate their “control over the situation”. If the patient is in need of individual treatment (orthopaedic surgeon, physiotherapist, psychologist) an appointment can be arranged by the hospital.

*Follow-up*

The somatic treatments and any eventual complications will be registered continuously. All included patients (randomized patients with risk factors and non-randomized controls) will be followed up at 6 and 12 months according to parameters below:

- Health related quality of life (questionnaires): SF-36 (generic, profile) and EQ-5D (generic, index), SMFA (disease specific).
- Ratings (Visual Analog Scale, VAS): pain, psychological well being, sense of control over the situation
- Self-reported recovery (one question): Do you feel recovered after your injury?
- Self-reported duration of sick leave

The primary outcome variables are self reported recovery, HRQoL compared to pre injury (retrospective rating) and Swedish normal population, and duration of sick leave. The PPS Risk prediction is related to the recovery rate in the total study population. Sample size calculation showed that 140 persons need to be randomized (80% power).

Ethical considerations

Is it ethically acceptable to ask patients with minor injuries questions about their background and how they feel, and then to offer some of them more psychosocial support than usual? Patient inconvenience due to questioning is of importance but deemed acceptable when considering the aim of improving the treatment of this patient group. Can an intervention program focusing on psychosocial factors be provocative for some patients? There is always a risk that some patients will find such a program provocative but since the program is supportive and does not aim to analyze the patients problems per se this risk should be low. Can the group sessions make some persons feel exposed? This is a risk, but we believe that aiming to create a positive atmosphere where no one is forced to talk will minimize problems with the group sessions. Psychiatric expertise is also available for consultation if necessary.

Clinical importance

It is often said that patients with minor injuries, including those injured in traffic accidents, recover in a couple of weeks and therefore no active follow-up is needed. However, our previous studies have shown that the recovery rate among patients with orthopaedic injuries is lower than generally expected and that some patients are at risk of non-recovery regardless of the injury or accident type. This study focuses on early identification and support of these individuals. If this randomized study shows our multidisciplinary support program to be effective then we have designed a practical “school” of 4 interactive sessions which can be implemented with low personnel costs into everyday healthcare

## Costs year 1

### Salaries

Study doctor,(Ph.D. student) 30% 205.500

Study nurse 50% 202.500

Anaesthetist 5% 45.000

Psychologist 5% 75.000

Physiotherapist 5% 35.000

### Other costs

Data management 20.000

Blood samples 10.000

Other (material, mailing costs) 10.000

### Total 603.000

## Grants year 1

Cancer och Trafikskadades Riksförbund 462.200

Försäkringsmedicinska Sällskapet 50.000

Personell resources

Sari Ponzer principal investigator, MD, associate professor, Karolinska Institute

Olof Nyrén co-investigator, MD, professor, Karolinska Institute

Carin Ottosson co-investigator, study doctor, MD, Ph.D. student, KI

*Staff at the Stockholm Söder Hospital, Karolinska Institute*

Tina Levander Study nurse, RN

Owe Landström MD, anaesthetist

Kjell Flodgren Psychologist

Martin Nordlander Physiotherapist
